# The impact of physical activity to the child’s quality of life: a bibliometric study

**DOI:** 10.12688/f1000research.18838.1

**Published:** 2019-05-16

**Authors:** Jernej Završnik, Peter Kokol, Helena Blažun Vošner

**Affiliations:** 1 Community Healthcare Center Dr. Adolf Drolc Maribor, Maribor, 2000, Slovenia; 2 Faculty of Electrical Engineering and Computer Science, University of Maribor, Maribor, 2000, Slovenia; 3 Faculty of Health and Social Sciences Slovenj Gradec, Slovenj Gradec, 2380, Slovenia

**Keywords:** Physical activity, Health related quality of life, Bibliometric mapping, Scientific landscape

## Abstract

**Background:** The application of bibliometrics in healthcare research is becoming popular, however at present it is still an under-researched area.

**Methods: **In our study we used a bibliometric technique called bibliometric mapping to visualize the published research regarding the influence of physical activity to children’s quality of life. The research was visualized in the form of both chronological and cluster science landscapes. Science landscapes, contrary to conventional reviews, capture the relationships between multiple topics and concepts, enabling the generation of “synthetic reviews”.

**Results:** Evolutionarily, three distinct research phases appeared, namely research on influence of physical activity on various chronic non-communicable diseases; research on quality of life and childhood diseases related to physical activity; and outcome-related research. The research consists of six main topics: asthmatic child and exercising, blood diseases, health-related quality of life, obesity and chronic diseases, childhood obesity and behaviour, and depression and health outcomes.

**Conclusions: **The study identified some research that may be helpful to general paediatricians whose everyday practice or research is not focused on physical activity and child’s quality of life, but wants to learn about the taxonomy of the topics, the most interesting discoveries, guidelines and practices and the state of the art in the field. It also revealed some hidden association, otherwise not easily identified, even by informed researchers and clinicians.

## Introduction

The role of bibliometrics in healthcare research has been excellently described by Lewison and Devey ^1^: “Bibliometrics is to scientific papers as epidemiology is to patients.” Indeed, in 1990 bibliometrics first became a medical subject heading. Consequently, the application of bibliometrics by health professionals to analytical, clinical, informational and academic areas of interest is becoming more extensive; however, at present it is an unexploited area of a fruitful research ^2^.

Interdisciplinary research concerning the influence of physical activity and sport on children’s quality of life is increasingly becoming more and more important. In their review paper Buttitta *et al*. ^3^ reported that several factors are associated with children’s quality of life; among them physical activity is one of the most important, especially in in those with pre-existing diseases. Various malignant diseases and anticancer therapy in children both drastically affect daily life activities, including high-performance sports. However, both random and non-random feasibility studies show positive effects of physical activity on clinical and psychosocial outcomes. Consequently, every effort should be made to maintain physical activity during paediatric cancer therapy ^4^. Comparable findings for congenital heart diseases, were reported by Dulfer *et al*. ^5^.

Since our research area interest is multidisciplinary, we had an opportunity to employ a bibliometric technique called bibliometric mapping. Bibliometric mapping visualizes academic research in the form of scientific landscapes ^6^. Science landscapes, contrary to conventional reviews, which focus on particular research questions, extract information at various levels and capture the relationship between multiple topics and concepts, creating “synthetic reviews”. Scientific landscapes are still relatively rarely used; however, those published have been well received by the scientific community ^7^.

## Methods

Bibliometrics could be defined as the application of mathematical, statistical and heuristic methods to scientific publications ^8^. Bibliometric mapping is a recent addition to techniques already used in bibliometrics in medicine ^2^. Bibliometric mapping aims to visualize different facets of literature production based on different co-occurrences (i.e. words, authors, organisations, journals) ^9^, co-citations ^10^ and bibliographical coupling ^11^. Bibliometric mapping can be automated using various software tools. Among open licence software tools, VOSviewer ^12^ is very versatile and easy to use ^13^. Both bibliometric mapping and VOSViewer have been successfully used in health related fields ^14^.

### Bibliographical dataset and corpus

The corpus was extracted from the Scopus bibliographical dataset, because of Scopus broad coverage of various journals, book chapters and conference proceedings on one hand and on the other hand because of Scopus’ extensive and easy to use search and analytical functions. The search was made on 12th January 2019 using the search keyword string child* AND ("physical activity" or "sport") AND "quality of life" in information source titles, abstracts and keywords for the whole period covered by Scopus.

### Data extraction and analysis

Publication year, source title and author's country of affiliation were extracted by Scopus services. Abstracts were exported as comma separated values (CSV) files to enable further analysis by the VOSviewer (V1.6.9) and Excel software (Version 2016). Cluster and chronological scientific landscapes were generated for all terms with an occurrence frequency larger than 30. Common words such as report, significance, trial, study and baseline were ignored using a customised thesaurus file (see Underlying data ^15^). Similarly, synonyms like body mass index and BMI were integrated into one term. The Attraction was set to 4, Min. Cluster size to 8, Resolution to 1.25; all other VOSViewer parameters were set to default values.

### Meta-synthesis

The popularity of a term (size of the term font and associated square) and relatedness between terms (terms located near each other are more related than those further apart) in both cluster and chronological scientific landscapes were analysed using meta-synthesis ^16^. An appropriate topic was determined for each cluster based on the analysis of most popular terms belonging to the cluster.

## Results

The search resulted in 2334 publications (1637 articles, 419 reviews, 66 conference papers, 50 editorials, 35 notes, 29 book chapters, 19 letters, 19 short communications and 24 other types of publications; see Underlying data ^15^). The most popular journals in which the above publications appeared are shown in Table 1. The most prolific journal was BMC Public Health with 31 publications. The highest ranked journal in the selected field was Pediatrics, with 21 publications (621 ^st^ place according to SCImago Journal Rank - SJR, which ranks within the top 2% of all source titles indexed by SciMago. Interestingly, all of the most prolific source titles are ranked in the top quarter of all journals. Their contribution to the total literature production on our topic of interest accounts for 12.4%. As expected most of the most prolific source titles are from the field of paediatrics, others relate to quality of life or from general areas. Surprisingly no source titles related to sport or physical activity were ranked among the most prolific source titles – the first (American Journal of Sports Medicine) is in 15 ^th^ place with 11 publications.

**Table 1.** The most prolific source titles on the topic of research, the influence of physical activity to children’s quality of life.

Source title	Number of publications
BMC Public Health	42
Quality of Life Research	39
Haemophilia	36
Pediatric Blood and Cancer	32
Pediatrics	27
Cochrane Database of Systemic Reviews	24
PloS One	20
BMC Pediatrics	19
Developmental Medicine and Child Neurology	17
Epilepsy and Behaviour	17
Health and Quality Of Life Outcomes	17
	∑ = 290 (12.4%)

The most productive countries are presented in Table 2. All of them have advanced heath, industrial and economic systems, high BDP and are also leading countries in research and development. The top 10 countries produced more than 83% of all publications, indicating that literature production is regionally centred.

**Table 2.** The 10 most productive countries on the topic of research, the influence of physical activity to children’s quality of life.

Country	Number of publications
United States	688
United Kingdom	240
Canada	223
Australia	204
Germany	166
Netherlands	125
Spain	113
France	97
Italy	95
Sweden	81
	∑ = 1951 (83.6%)

### The trends in research literature production and evolution of research topics

**Figure 1.**
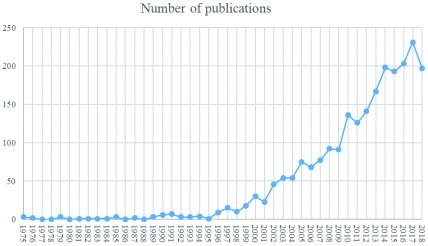
The dynamics of literature production on the topic of research, the influence of physical activity to children’s quality of life.

Shen *et al*. ^17^ proposed a model of science discipline development based on literature production dynamics. Following their example, a graph was constructed ( Figure 1) which reveals three distinguished phases in the production of publications regarding influence of physical activity to children’s quality of life, namely:

- initial phase in the period 1975–1989 when publications were scare, most three publications per year;
- initiation phase in the period 1990–1999 when number of publications linearly increased from 6 to 23; and
- exponential growth phase in the period 2000–2018, when production reached its peak in 2017 with 231 publications.

**Figure 2.**
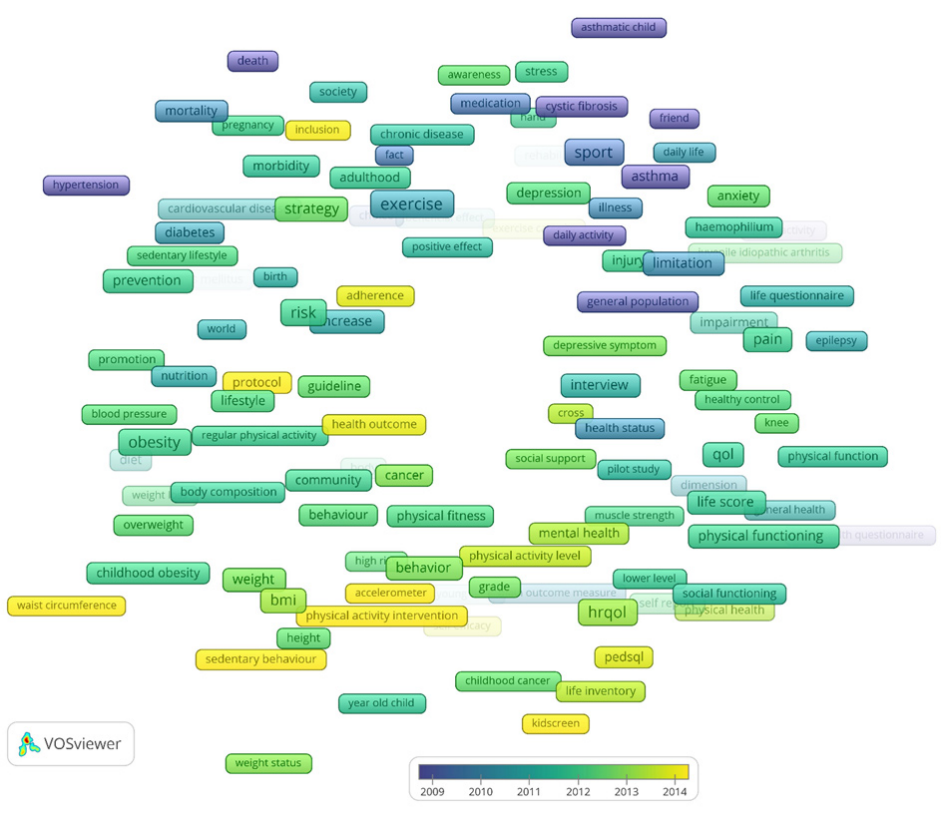
The chronological science landscape of the influence of physical activity to children’s quality of life.

Figure 2 presents the term map on the topic of research, the influence of physical activity to children’s quality of life. According to the figure the research development went through three main phases, namely:

1. **Research on the influence of physical activity on non-communicable diseases** (approx. period from 1975 to 2011 – violet and blue colours). The associations between (1) *hypertension, diabetes, cardiovascular diseases*, *mortality, exercise* and positive/ *beneficial* effects, and (2) *asthma, cystic fibrosis, medication, illness, sport* and *daily activity*, were the main stream of research in the first phase.
2. **Research on frequent childhood diseases and physical activity** (green and light blue colours – approx. period from 2012 to 2013). The research was focused on associations between (1 *) chronic diseases, society, pregnancy, birth, sedentary life style, prevention strategies*, and *risk increase* (2), nutrition, promotion, blood pressure, obesity, physical activity and, fitness and (3) *anxiety, depression, stress, juvenile idiopathic arthritis, haemophilia, injury* and *health status*.
3. **Measuring quality of life** (light green and yellow colours – state-of-the-art research). The research in the most recent period is concerned with association between (1) *inclusion*, *adherence*, *protocol, cancer* and *health outcomes,* (3) *sedentary behaviour*, *weight status, physical activity level* and *physical activity interventions* and (4) *quality of life indicators, physical functioning and physical health.*

**Figure 3.**
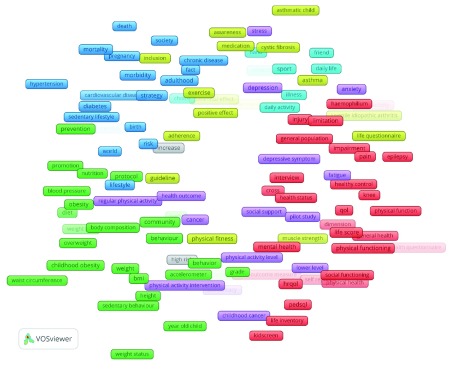
The cluster science landscape on the topic of research, the influence of physical activity to children’s quality of life.

The cluster science landscape ( Figure 3) consists of six clusters. Meta-synthesis revealed six topics, as shown in Table 3.

**Table 3.** Topics derived from the cluster science landscape.

Topic	Cluster colour	Popular terms
Childhood obesity and behaviour	Green	Childhood obesity, BMI, behaviour, community, obesity, weight
Chronic diseases and lifestyle	Blue	Diabetes, cardiovascular diseases, hypertension, chronic diseases, risk, life style, morbidity
Asthmatic child and exercising	Yellow	Asthma, asthmatic child, exercise, physical fitness, positive effect, awareness, medication
Health-related quality of life and physical and social functioning	Red	Health-related quality of life (HRQOL), paediatric quality of life ((PEDSQL), social functioning, physical functioning, physical health, life inventory, Kidscreen, pain
Illness and sport	Light blue	Illness, daily activity, sport, friend
Depression and regular physical activity	Violet	Depression, anxiety, stress, fatigue, physical activity intervention, health outcome, social support

## Discussion

It is interesting to note some details evident from the derived topics above and associations between terms:

- *The quantity of research concerning physical activity in relation to asthma, but not only as a single disease, but also in combination with obesity and diabetes.* Di Genova *et al.* ^18^ claim that growing evidence shows the existence of an “obese asthma” phenotype characterised by difficult-to-manage asthma. Additionally, Atay and Berket ^19^ show that obesity results in various co-morbidities in children and adolescent.
- *The increasing incidence of psychosomatic diseases and the beneficial effects of physical activity.* Hrafnkelsdottir *et al*. ^20^ reports that less screen time and more intense physical activity lowers the risk of mental health problems.
- *The broadness of the spectrum of diseases related to child’s quality of life and physical activity.* Studies highlighted that children with overweight/obesity and related co-morbidities and lifestyle behavioural risk factors, had significantly lower healthy-related quality of life ^21, 22^.
- *The introduction of modern technology such as various sensors in both research and practice supporting the empirical research in areas that were previously very subjective.* Traffic-related air pollution and noise may lead to adverse health outcomes, including increased blood pressure, myocardial infarction, and respiratory health in paediatric population. Measuring the physical activity and environmental factors revealed that such measurements can lead to better understanding of the relation between above factors ^23^. Another study investigated the use of a carbohydrate intake based on continuous glucose monitoring trends during physical activity of children with diabetes to objectively asses the association between these two variables ^24^.
- *The state-of-the-art efforts in the development of specific child’s quality of life indices and instruments to measure their relatedness to other paediatric indices.* Moghaddaszadeh *et al*. ^25^ measured the paediatric quality of life indicators and their relations to enjoyment levels and physical attractiveness.
- *The relationship between acute lymphoblastic leukaemia and haemophilia, and physical activity.* Acute lymphoblastic leukaemia treatment in children can result in muscle weakness and motion limitations. A study showed that active dorsiflexion range of motion combined with physical activity had a positive correlation with strength/agility standard score ^26^. Haemophilia management recommends physical activity in children with haemophilia. In a study researchers adapted and validated the adult Haemophilia & Exercise Project-Test-Questionnaire (HEP-Test-Q) for children aged 6–17 years, reformulated questionnaire items to make them understandable to children ^27^.

## Conclusion

Using bibliometric mapping we created two scientific landscapes on the topic of research, the influence of physical activity to children’s quality of life. To the best of our knowledge, this is the first such attempt in the paediatric field. We identified six distinct topics and also visualised the chronological aspect of the research literature production. The study revealed some knowledge that might be helpful to an “outsider” who wants to learn about the taxonomy of the topics, the most interesting discoveries, guidelines and practices and the state of the art in the field. It can also help the “seasoned insiders” better understand more specialised research including that is out of their immediate scope of interest. Additionally, it can reveal hidden facts, not easily identified, even by informed researchers and clinicians.

## Data availability

### Underlying data

Open Science Framework: Physical activity and quality of life in children. https://doi.org/10.17605/OSF.IO/WXSQE ^15^

This project contains the following underlying data:

- Map.txt (Map file for VOSViewer software)
- Network.txt (Network file for VOSViewer software)
- Scopus1.csv (Extracted articles from Scopus 2011–2019)
- Scopus2.csv (Extracted articles from Scopus 1975–2010)
- Thesaurus_terms.txt (Customised thesaurus file defining terms to be omitted and synonyms)

Data are available under the terms of the Creative Commons Zero "No rights reserved" data waiver (CC0 1.0 Public domain dedication).

## References

[1] Lewison G Devey ME: Bibliometric methods for the evaluation of arthritis research. *Rheumatology (Oxford).* 1999;38(1):13–20. 10.1093/rheumatology/38.1.13 10334677

[2] Alfonzo PM Sakraida TJ Hastings-Tolsma M: Bibliometrics: Visualizing the impact of nursing research. *Online J Nurs Inform.* 2014;18(1). Reference Source

[3] Buttitta M Iliescu C Rousseau A: Quality of life in overweight and obese children and adolescents: a literature review. *Qual Life Res.* 2014;23(4):1117–39. 10.1007/s11136-013-0568-5 24249217

[4] Götte M Taraks S Boos J: Sports in pediatric oncology: the role(s) of physical activity for children with cancer. *J Pediatr Hematol Oncol.* 2014;36(2):85–90. 10.1097/MPH.0000000000000101 24390449

[5] Dulfer K Helbing WA Duppen N: Associations between exercise capacity, physical activity, and psychosocial functioning in children with congenital heart disease: a systematic review. *Eur J Prev Cardiol.* 2014;21(10):1200–15. 10.1177/2047487313494030 23787793

[6] Kokol P Blažun Vošner H Železnik D: *Clinical Simulation in Nursing*: A Bibliometric Analysis after Its Tenth Anniversary. *Clin Simul Nurs.* 2017;13(4):161–7. 10.1016/j.ecns.2016.11.007

[7] Lee CISG, Felps W Baruch Y: Toward a taxonomy of career studies through bibliometric visualization. *J Vocat Behav.* 2014;85(3):339–51. 10.1016/j.jvb.2014.08.008

[8] Bellis ND: Bibliometrics and Citation Analysis: From the Science Citation Index to Cybermetrics. Lanham, Md: Scarecrow Press;2009;450 Reference Source

[9] Chen X Chen J Wu D: Mapping the Research Trends by Co-word Analysis Based on Keywords from Funded Project. *Procedia Comput Sci.* 2016;91:547–55. 10.1016/j.procs.2016.07.140

[10] Small H: Co-citation in the scientific literature: A new measure of the relationship between two documents. *J Am Soc Inf Sci.*Wiley Online Library [Internet]. [cited 2019 Apr 5].1973;24(4):265–269. 10.1002/asi.4630240406

[11] Biscaro C Giupponi C: Co-authorship and bibliographic coupling network effects on citations. *PLoS One.* 2014;9(6):e99502. 10.1371/journal.pone.0099502 24911416 PMC4049820

[12] van Eck NJ Waltman L Noyons EC: Automatic term identification for bibliometric mapping. *Scientometrics.* 2010;82(3):581–96. 10.1007/s11192-010-0173-0 20234767 PMC2830586

[13] Blažun H Kokol P Vošner J: Research literature production on nursing competences from 1981 till 2012: A bibliometric snapshot. *Nurse Educ Today.* 2015;35(5):673–9. 10.1016/j.nedt.2015.01.002 25616510

[14] Kokol P Završnik J Blažun Vošner H: Bibliographic-Based Identification of Hot Future Research Topics: An Opportunity for Hospital Librarianship. *J Hosp Librariansh.* 2018;18(4):315–322. 10.1080/15323269.2018.1509193

[15] Kokol P: Physical activity and quality of life in children.2019 10.17605/OSF.IO/WXSQE

[16] Bondas T Hall EO: Challenges in approaching metasynthesis research. *Qual Health Res.* 2007;17(1):113–21. 10.1177/1049732306295879 17170249

[17] Shen J Yao L Li Y: Visualizing the history of evidence‐based medicine: A bibliometric analysis. *J Am Soc Inf Sci Technol.*Wiley Online Library [Internet]. [cited 2019 Apr 5].2013;64(10):2157–2172. 10.1002/asi.22890

[18] Di Genova L Penta L Biscarini A: Children with Obesity and Asthma: Which Are the Best Options for Their Management? *Nutrients.* 2018;10(11): pii: E1634. 10.3390/nu10111634 30400197 PMC6267365

[19] Atay Z Bereket A: Current status on obesity in childhood and adolescence: Prevalence, etiology, co-morbidities and management. *Obes Med.* 2016;3:1–9. 10.1016/j.obmed.2016.05.005

[20] Hrafnkelsdottir SM Brychta RJ Rognvaldsdottir V: Less screen time and more frequent vigorous physical activity is associated with lower risk of reporting negative mental health symptoms among Icelandic adolescents. *PLoS One.* 2018;13(4):e0196286. 10.1371/journal.pone.0196286 29698499 PMC5919516

[21] Hoare E Crooks N Hayward J: Associations between combined overweight and obesity, lifestyle behavioural risk and quality of life among Australian regional school children: baseline findings of the Goulburn Valley health behaviours monitoring study. *Health Qual Life Outcomes.* 2019;17(1):16. 10.1186/s12955-019-1086-0 30658630 PMC6339321

[22] Yuen KC Koltowska-Häggström M Cook DM: Clinical characteristics and effects of GH replacement therapy in adults with childhood-onset craniopharyngioma compared with those in adults with other causes of childhood-onset hypothalamic-pituitary dysfunction. *Eur J Endocrinol.* 2013;169(4):511–9. 10.1530/EJE-13-0280 23904277

[23] Leaffer D Wolfe C Doroff S: Wearable Ultrafine Particle and Noise Monitoring Sensors Jointly Measure Personal Co-Exposures in a Pediatric Population. *Int J Environ Res Public Health.* 2019;16(3): pii: E308. 10.3390/ijerph16030308 30678120 PMC6388247

[24] Burckhardt MA Chetty T Smith GJ: Use of Continuous Glucose Monitoring Trends to Facilitate Exercise in Children with Type 1 Diabetes. *Diabetes Technol Ther.* 2019;21(1):51–5. 10.1089/dia.2018.0292 30620642

[25] Moghaddaszadeh A Jamnik V Belcastro AN: Characteristics of children’s physical activity during active play. *J Sports Med Phys Fitness.* 2018;58(4):369–76. 10.23736/S0022-4707.16.06704-9 27735889

[26] Tanner LR Hooke MC: Improving body function and minimizing activity limitations in pediatric leukemia survivors: The lasting impact of the Stoplight Program. *Pediatr Blood Cancer.* 2019;66(5):e27596. 10.1002/pbc.27596 30609245

[27] von Mackensen S Hilberg T Valentino LA: Validation of the Haemophilia & Exercise Project-Test-Questionnaire (HEP-Test-Q)-An instrument for the assessment of subjective physical functioning in children with haemophilia. *Haemophilia.* 2018;24(6):888–95. 10.1111/hae.13533 30004619

