# Supplementary material for: The impact of physical activity to the child’s quality of life: a bibliometric study
Source: F1000Res. 2019 May 16;8:672. [Version 1] doi: 10.12688/f1000research.18838.1 (PMC6720035; doi:10.12688/f1000research.18838.1)
Supplement: Peer review history

# Peer review: Reviewer response for version 1

**DOI:** 10.5256/f1000research.20643.r52912

**Published:** 2019-09-02

**Authors:** Julije Mestrovic

**Affiliations:** 1 National Paediatric Society of Croatia/Medical School of Split, University Hospital of Split, Split, Croatia

The work is interesting for readers and should be indexed. The following should be done first:

1. In the discussion, explain briefly the possible relationships between illness and physical activity. Namely, it is important to note what are the mechanisms of how physical activity can affect disease, but also vice versa, in a wide range of conditions, from asthma to air pollution.
2. In discussion, avoid overlapping conditions (asthma and obesity).

I confirm that I have read this submission and believe that I have an appropriate level of expertise to confirm that it is of an acceptable scientific standard, however I have significant reservations, as outlined above.

# Peer review: Reviewer response for version 1

**DOI:** 10.5256/f1000research.20643.r50557

**Published:** 2019-07-25

**Authors:** Manuel Jesus Cobo

**Affiliations:** 1 Department of Computer Science and Engineering, University of Cádiz, Cádiz, Spain

The manuscript develop a science mapping analysis of the physical activity on children and the effect in their quality of life. To do that, VOSViewer software tool is employed.

 In general, the paper is well organised and written. Moreover, the software tool is appropriate for this analysis and authors use it properly.

 In what follows, some comments and suggestions are listed:

- I miss some references to the origins of co-word analysis, science mapping, or generally, science of science. Authors focus too much on bibliometrics on health, but there are important references out of this field. Moreover, authors should cite some recent bibliometric studies developed in health, for example in cancer, rehabilitations, etc.
- The reference to VOSViewer is incorrect. Author should cite the paper where the software is presented.
- Figure 2 is confusing. Authors should provide other kind of visualisation. Also, authors should describe better the conceptual evolution of this research field.
- Discussion should be better described. At this moments it is just a sequence of itemises. Author should provided a clear speech.
- Conclusions are just a summary of the paper.

I confirm that I have read this submission and believe that I have an appropriate level of expertise to confirm that it is of an acceptable scientific standard, however I have significant reservations, as outlined above.

# Peer review: Reviewer response for version 1

**DOI:** 10.5256/f1000research.20643.r49710

**Published:** 2019-06-17

**Authors:** Zachi Grossman

**Affiliations:** 1 Pediatric clinic, Maccabi Healthcare Services, Tel Aviv, Israel

I find this manuscript interesting since it gives a new perspective on the literature.

 I would like to propose the following changes in order to improve the conclusions:

1. Single papers on blood diseases - haemophilia and leukaemia - are not enough to draw conclusions from. Therefore, I would tend to disregard these papers both in the discussion and in the abstract.
2. I am not so convinced in the existence of three distinguished time phases. I would prefer a two time phases division: before and after 1995.

I confirm that I have read this submission and believe that I have an appropriate level of expertise to confirm that it is of an acceptable scientific standard.
